# Simulating system dynamics of the HIV care continuum to achieve treatment as prevention

**DOI:** 10.1371/journal.pone.0230568

**Published:** 2020-03-19

**Authors:** Margaret R. Weeks, David W. Lounsbury, Jianghong Li, Gary Hirsch, Marcie Berman, Helena D. Green, Lucy Rohena, Rosely Gonzalez, Jairo M. Montezuma-Rusca, Seja Jackson

**Affiliations:** 1 Institute for Community Research, Hartford, Connecticut, United States of America; 2 Department of Epidemiology and Population Health, Albert Einstein College of Medicine, Montefiore Medical Center, Bronx, New York, United States of America; 3 Creator of Learning Environments, Wayland, Massachusetts, United States of America; 4 Department of Internal Medicine, University of Connecticut Health Center, Farmington, Connecticut, United States of America; 5 St. Francis Hospital and Medical Center, Burgdorf Health Center, Hartford, Connecticut, United States of America; Ohio State University, UNITED STATES

## Abstract

The continuing HIV pandemic calls for broad, multi-sectoral responses that foster community control of local prevention and care services, with the goal of leveraging high quality treatment as a means of reducing HIV incidence. Service system improvements require stakeholder input from across the care continuum to identify gaps and to inform strategic plans that improve HIV service integration and delivery. System dynamics modeling offers a participatory research approach through which stakeholders learn about system complexity and about ways to achieve sustainable system-level improvements. Via an intensive group model building process with a task force of community stakeholders with diverse roles and responsibilities for HIV service implementation, delivery and surveillance, we designed and validated a multi-module system dynamics model of the HIV care continuum, in relation to local prevention and care service capacities. Multiple sources of data were used to calibrate the model for a three-county catchment area of central Connecticut. We feature a core module of the model for the purpose of illustrating its utility in understanding the dynamics of treatment as prevention at the community level. We also describe the methods used to validate the model and support its underlying assumptions to improve confidence in its use by stakeholders for systems understanding and decision making. The model’s generalizability and implications of using it for future community-driven strategic planning and implementation efforts are discussed.

## Introduction

As the HIV pandemic approaches four decades, nations, states, and communities increasingly seek broad, multi-sectoral responses to prevent new infections while caring for those infected in order to achieve the goal of controlling the epidemic [1–3]. Community health outcomes depend on a coordinated, highly effective local HIV testing, treatment, and care service system to find, treat, and maintain viral suppression in all people living with HIV (PLWH) [4, 5]. To reduce or eliminate the epidemic, this healthcare delivery and support system must address the “treatment cascade,” in which PLWH fall out of care [6–8], and effectively stop new infections through both primary prevention and treatment-as-prevention (TasP) [9, 10].

System improvements require stakeholders from across the care continuum to identify service gaps and develop and carry out strategic plans to improve HIV service integration and delivery [11–13]. However, such plans may suffer from stakeholders’ incomplete “mental models,” that is, their internal conceptual representation of the HIV care system [14, 15], and their insufficient recognition of system complexity and limited understanding of system dynamics that affect population health outcomes [16, 17]. They need tools to help them recognize interdependence, engage together to envision possible solutions, and plan the most promising set of strategies to avoid misdirecting limited resources or proposing simple solutions for complex problems.

Participatory system dynamics (SD) modeling brings stakeholders together to examine complex problems at the community level [18, 19]. The approach has been used to examine various public health concerns [20–24], including HIV/AIDS [25–27]. Participatory SD modeling engages stakeholders in an iterative “systems thinking” process that contributes to model building by diagraming, critiquing, seeking data to quantify and calibrate, and simulating interactive systems-level dynamics with the aid of computer tools [28–30]. It also allows community stakeholders to learn about system complexity and service gaps from each other and through the modeling process, in order to identify mechanisms likely to lead to or impede system improvements. SD simulation models that have been developed and validated through a participatory stakeholder model building process offer conceptual, methodological, and analytical tools to support identification of system-level strategies to achieve public health goals [31–34]. Stakeholders can generate hypothetical single, multiple, or sequential evidence-based and locally generated interventions and other actions expected to attain optimal outcomes. They can then test these strategies virtually through simulation before expending effort and resources to implement them [20, 27, 35].

**Fig 1:**
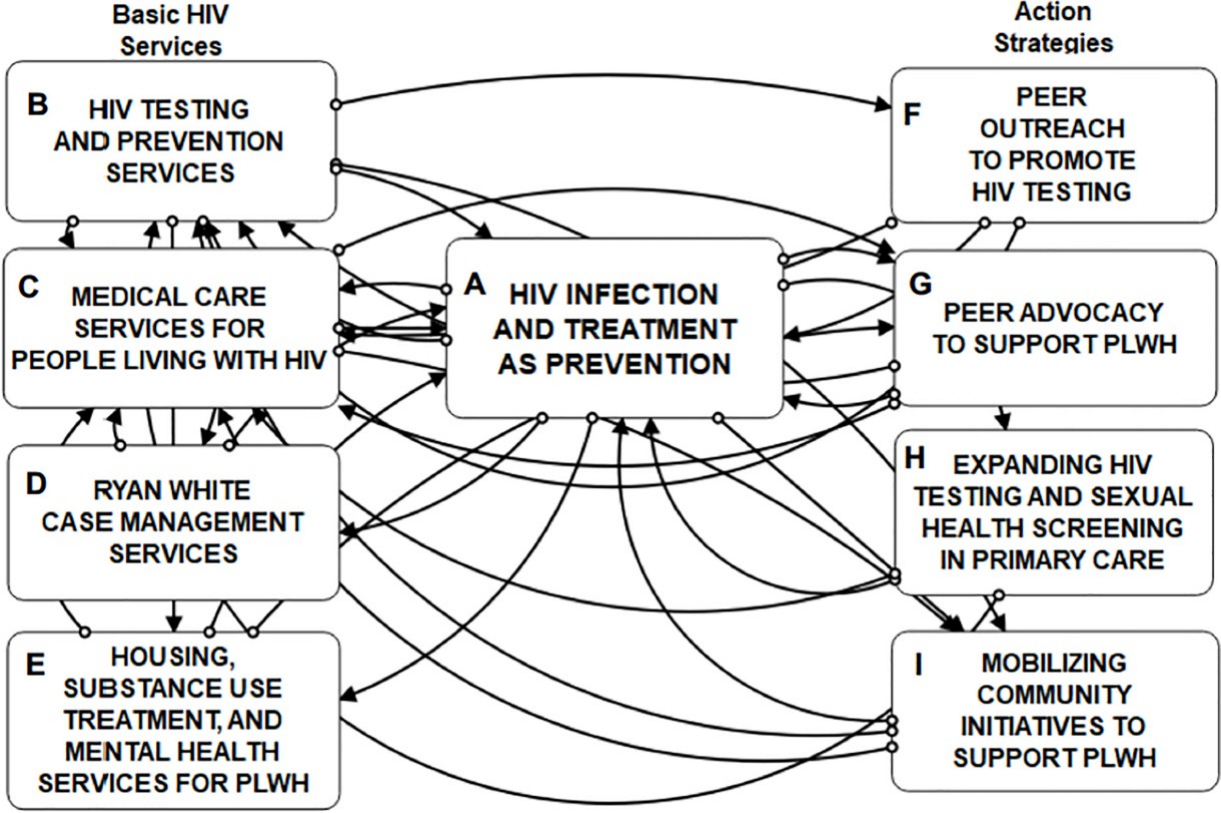
Modules comprising the system dynamics model of the HIV care continuum. Treatment as Prevention (Module A, center); Basic Services (Modules B-E, left); Action Strategies (Modules F-I, right).

We used group model building (GMB) [36–39] to develop, calibrate, and validate a computational tool [40] representing the HIV health and social services care continuum (CC). The overarching purpose of the tool is to use it to inform effective, community-driven ways of leveraging TasP, which can be visualized as an omnipresent (although not always apparent) feedback loop. In this loop, improvements in viral suppression among PLWH serve to decrease likelihood of HIV exposure and infection, theoretically to the point of any further new cases in the total population. Our resultant model is organized into nine interdependent modules (A-I, see **Table 1 and Fig 1**). Modules that illustrate interdependencies among Basic Services (such as HIV medical care and access to substance use treatment, Modules B-E) and Action Strategies (such as peer outreach and community initiatives, Modules F-I) shape patterns of HIV infection, diagnosis, access to care, and viral suppression in a given community or targeted catchment area over time (HIV Infection and Treatment as Prevention, Module A).

**Table 1:** Modules of the HIV Care Continuum (CC) System Dynamics (SD) simulation model.

Module Name	Module Type	Module Description
A. HIV Infection and Treatment as Prevention (TasP)	TasP	This is the central HIV test, treat, and retention in care module representing the stages of the care continuum and the “treatment cascade,” from HIV exposure and infection, to diagnosis, linkage to care, initiation of anti-retroviral treatment (ART), and viral suppression, or lost to care and mortality. In this module, the effectiveness of TasP impacts the rate of new HIV infections at the population level, thereby representing the primary TasP balancing feedback loop.
B. HIV Testing and Prevention Services	Basic Service	This module aggregates all community HIV testing and prevention programs including: “General HIV Testing in Low Prevalence Settings,” “General HIV Testing in High Prevalence Settings,” “Targeted HIV Testing Services” to reach high-risk groups (who often do not use other testing services), “PrEP Referral and Implementation for HIV-negative people at high risk,” and “Partner Services Referral for HIV-positive People” to seek their partners for HIV testing. People who test HIV-positive also link to the “Medical Care Services” module to enter medical care for HIV.
C. Medical Care Services for People Living with HIV (PLWH)	Basic Service	This module links newly diagnosed people with HIV to medical care and simulates their repeated medical appointments, missed appointments, and lost to care dynamics. Outcomes of Medical Care Services link to the central “HIV TasP” module to increase viral suppression in PLWH.
D. Ryan White Case Management Services	Basic Service	This module represents case management needs among PLWH and provision and limitations of Ryan White (RW) case management services. Unmet case management needs link to the “Medical Care Services” module as an effect on the linked to care and lost to care rates.
E. Housing, Substance Use Treatment, & Mental Health Services for PLWH	Basic Service	This module includes three designated service models, including: 1) “Housing Needs and Services,” 2) “Substance Use Treatment Needs and Services,” and 3) “Mental Health Care Needs and Services.” Unmet needs for these services link to the “Medical Care Services” module to affect the lost to care rate.
F. Peer Outreach to Promote HIV Testing	Action Strategy	This module represents a program to increase the community HIV testing rate by engaging people who get tested for HIV to recruit their peer network members to get tested as well. Effects of this program increase the monthly HIV testing rate in the “HIV Testing and Prevention” module in all three test settings.
G. Peer Advocacy to Support PLWH	Action Strategy	This module represents a program to train and deploy Peer Advocates (sometimes called Peer Navigators) to support and empower other PLWH to access and stay in medical care and adhere to their HIV medications. Effects of this program link to the “Medical Care Services” module to reduce the lost to care rate.
H. Expanding HIV Testing & Sexual Health Screening in Primary Care	Action Strategy	This module represents expanded HIV testing and comprehensive sexual health screenings by primary care providers to their patients. Effects of this action strategy increase HIV testing in all general testing settings and increase PrEP implementation and potential uptake.
I. Mobilizing Community Programming to Support PLWH	Action Strategy	This module represents implementation of programs to reach community members broadly as well as to target high risk individuals and families of PLWH with HIV information and supportive programs. The module simulates impacts of those programs to increase community-level HIV knowledge, and to reduce HIV related stigma and medical mistrust in the community. Effects of these programs link to the “Medical Care Services” module to reduce the lost to care rate, and to the “HIV Testing and Prevention” module to increase the HIV testing rate in all test settings.

This article provides an overview of the full HIV CC SD model, with a more detailed focus on the structure and behavior of Module A, and implications of using the full model for future community-driven strategic planning and implementation efforts. Full documentation of each module’s equations, parameter estimates, and other supportive materials are available at https://github.com/mweeks56/ICR_HIV_Care_SDM (the project’s on-line repository) and at the protocols website dx.doi.org/10.17504/protocols.io.bcm6iu9e.

## Methods

### Overview of system dynamics (SD) model building

SD models specify interdependencies among key constructs that define complex feedback structures for a given problem of interest. Feedback structures, or loops, are of two types, namely, reinforcing, which generate an acceleration effect, and balancing, which generate a buffering effect or equilibrium. SD models explicitly identify time delays that underlie systems-level processes, which often help explain problematic or counterintuitive patterns of system behavior (outcomes) [41, 42].

SD modeling projects often begin by sketching qualitative causal loop diagrams (CLD) [37, 43] and by formulating a quantitative computational model, or “scoping” model [40], applying stakeholders’ knowledge of the focal problem and relevant available quantitative and qualitative secondary sources of evidence. CLDs are used to develop stock-and-flow diagrams, which are coded using graphical software (we used Stella Architect 1.9.4®). Stock-and-flow diagrams hold a set of algebraic and differential equations, with key parameter estimates chosen from the highest quality empirical and historical data [40]. Stocks specify accumulations of units, such as people, things, or information. Flows specify rates of change of units from one stock, or condition or state, to another.

The behavior of the model is assessed, in part, by comparing simulated output to major “reference modes,” which are typically historical trends and future anticipated outcomes over a specific time horizon for important constructs in the model (e.g., HIV incidence or prevalence). Reference modes may also depict hypothesized patterns or behaviors over time for unmeasured constructs, such as the level of stigma in the community. Once the model is deemed adequate in terms of its scope and purpose by participating stakeholders, and once it passes fundamental structural and behavioral (pattern) tests, it can be used to compare and contrast various simulated policy or intervention solutions [40].

### Study design

Our research team conducted a 3-year study (R01-MH103176; 2015–2018) using mixed methods and GMB to examine barriers and facilitators to HIV prevention and care in a three-county area in central Connecticut [39]. Study methods included group and individual interviews with HIV medical and social service providers and PLWH about barriers and facilitators to HIV care delivery, and repeated surveys (baseline, 6-month, 12-month) with a cohort of PLWH (N = 200) and high risk uninfected persons (N = 56) about their HIV service utilization and the community context that affects their interactions with the HIV CC. They also included a series of GMB sessions (described below) to conceptualize and design the SD model of the HIV CC system. Data from qualitative interviews and surveys informed several components of the SD model structure [39]. A concurrent 2-year study (R21-MH110335; 2016–2018) provided resources to translate stakeholders’ visual models (CLDs) into validated stock-and-flow structures [15, 44, 45], which formed our full computational model of the HIV CC [39].

To design and test the model structure, we engaged a 25-member group representing HIV community organizations in the region, public and private health institutions, and PLWH in an iterative, GMB process of systems thinking and SD model development. This “SD Modeling Task Force” included 5 HIV-positive peer advocates, 5 direct medical service providers, 4 case managers, 7 directors of community-based service organizations or HIV prevention or support programs, 3 public health HIV program directors at the state and city levels, and a local pharmaceutical company community liaison. We conducted an iterative sequence of sixteen 2½-hour monthly GMB sessions over an 18-month period (2017–2018). Stakeholders were tasked with critiquing and mapping the regional HIV CC system using SD visual modeling and qualitative narrative [39] to identify both effective services and gaps or disconnects in service delivery and to inform model parameterization. Throughout the SD model development period, we retained all but two Task Force members, and session attendance ranged from 70% - 100% [39].

All protocols for the protection of human subjects in research were followed during these studies, which received full review and approval by the Institutional Review Board of the Institute for Community Research. All study participants and the SD Modeling Task Force members provided written informed consent before initiating research activities on this study.

### Model conceptualization, calibration and validation

#### Designing and calibrating the model

Prior to initiating the GMB sessions with the SD Modeling Task Force, the project’s primary SD modeler (second author) developed an initial computational (scoping) model of the HIV treatment cascade to represent the HIV CC for the purpose of demonstration and to initiate the modeling effort. The structure of the scoping model included the stages of the care continuum, from HIV incidence and undiagnosed status, to testing and diagnosis, linkage of PLWH to medical care, engagement in care and on ART, and achievement of viral suppression. This initial scoping model was parameterized using data from the CT Department of Public Health (DPH) 2015 HIV Surveillance Report [46] and calibrated to reproduce the subsequent two years of epidemiological patterns in the three-county area.

During GMB sessions, we distilled and refined Task Force members’ narratives and concept maps to specify and integrate services, programs, and other system contributors [39]. The structure of the initial HIV CC scoping model was expanded to incorporate the commonly available healthcare and social service resources (Basic Services, Modules B-E in Table 1 and Fig 1) and potential or hypothesized community intervention programs (Action Strategies, Modules F-I in Table 1 and Fig 1) that contribute to system dynamics and health outcomes. After modifying the initial scoping model and updating initial parameters, we chose start and end points for a 60-month time horizon (5 years; corresponding to calendar months t_0_ = 01/01/2018 and t_60_ = 01/01/2023). Stakeholder input and deliberation was elicited regarding the values assigned to initial parameter estimates of stocks and flows, as well as other ancillary variables or cofactors used to formulate model equations. To demonstrate effective SD model performance, we compared base case scenario simulated output for the prior calendar year (2017; t_-12_ = 01/01/2017 to t_-1_ = 12/31/2017) with reported trends over the same period in the catchment area (described more fully below). This technique was also used to resolve any computational anomalies due to initial parameter values [47].

#### Model validation process

SD model validation occurs through an iterative process of model conceptualization, calibration, and simulation, via cycles of deliberation, data input, review, and decision-making by key stakeholders [24, 45, 48–53]. Our iterative model development and revision process focused on four types of model validity.

Structural validation determines that the model has been formulated with accuracy and adequately represents the model developers’ conceptual description of the system. This was achieved during iterative GMB sessions for model conceptualization and revision, with support from the theoretical and empirical literature. Specifically, as we developed the model designs of each of the subsystems (modules in Table 1), which we derived from Task Force narratives and from known relationships supported by extant literature on TasP, we presented them at subsequent meetings for Task Force member deliberation and feedback on model components and the causal relationships among those elements. Our research team also iteratively rechecked variable parameters and formulations for inaccuracies, redundancies, and omissions to discuss and resolve them during weekly modeling sessions.

Behavioral validation involves assessing the model’s simulated behavior and assuring that it has sufficient accuracy for its intended purpose over the scope of its intended applicability. Behavioral validity supports the model’s credibility with stakeholders and others. This was initiated during the GMB process by comparing simulated reference modes (output graphs) with epidemiological trend data to assure correct initial calibration to the local context. We examined preliminary simulation runs iteratively in sessions with the SD Modeling Task Force to check and re-check the extent to which the model’s behavior (simulated output) conformed to key assumptions and sources of evidence guiding the study [39]. For example, we compared simulated output of new infection rates to historical 5-year trends in the catchment area, as well as state-wide trends in the epidemic as appropriate, both of which have plateaued into a steady state during the past 4–6 years. Other TasP outcomes calibrated to replicate effectiveness of the regional HIV CC included the numbers of PLWH engaged in and lost to care over time and the percent virally suppressed. Parameter estimates derived from these sources were evaluated by running the model. (We present and discuss this comparison of our base case simulation to epidemiological reference modes more fully below.)

Construct validation involves assessing the extent to which the model is supported by relevant theories, frameworks and assumptions underlying the problem of interest. In this case, we relied on a growing body of literature regarding the concept of TasP, which posits that infections at the community level can be reduced by identifying all PLWH in the community and providing them continuous ART to achieve and permanently maintain viral suppression, thereby reducing “community viral load” [54, 55]. This literature focuses on community, organizational, and individual level factors that facilitate moving PLWH quickly through all stages of the HIV CC (i.e., minimizing delays in achieving viral suppression), and retaining them in medical care over time [4, 8, 56, 57].

Data validation ensures that the data (and/or special knowledge) necessary for model formulation and calibration (capturing past trends), evaluation (predicting future trends), and application (virtual experiments and policy analyses) are adequate and reliable for the intended purpose of the model. Use of primary and secondary data, literature review, and stakeholders’ best estimates are common practices in the parameterization of SD model components [58]. This was assured through our use of key regional epidemiological and health services utilization data, including CT DPH surveillance data (e.g., state reportable viral load counts of PLWH in care, treatment cascade data, linked to care and late testing rates, etc. [46, 59]) and Ryan White health services data (e.g., case management, housing, and transportation needs, and gaps in substance use treatment and mental health services [60]). These sources generally use metrics commonly used state-wide and nationally [61–63], thereby increasing generalizability and potential applicability of the SD model beyond the geographic region in which it was developed.

We also analyzed qualitative and quantitative data from our concurrent study to identify variables for the model and parameters for some variables. For example, data from the study’s cohort provided estimates for some transition rates of PLWH through care continuum stages and global measures of perceived external stigma. Task Force members’ experiential knowledge of the service delivery process and other contextual factors that have no known or standard metrics provided a means to parameterize initial (starting) values of other variables, such as time delays, caseloads, missed appointment rates, and community attitudes like medical mistrust and HIV knowledge, among others.

## HIV Infection and treatment as prevention (Module A)

### Causal Loop Diagram (CLD)

**Fig 2:**
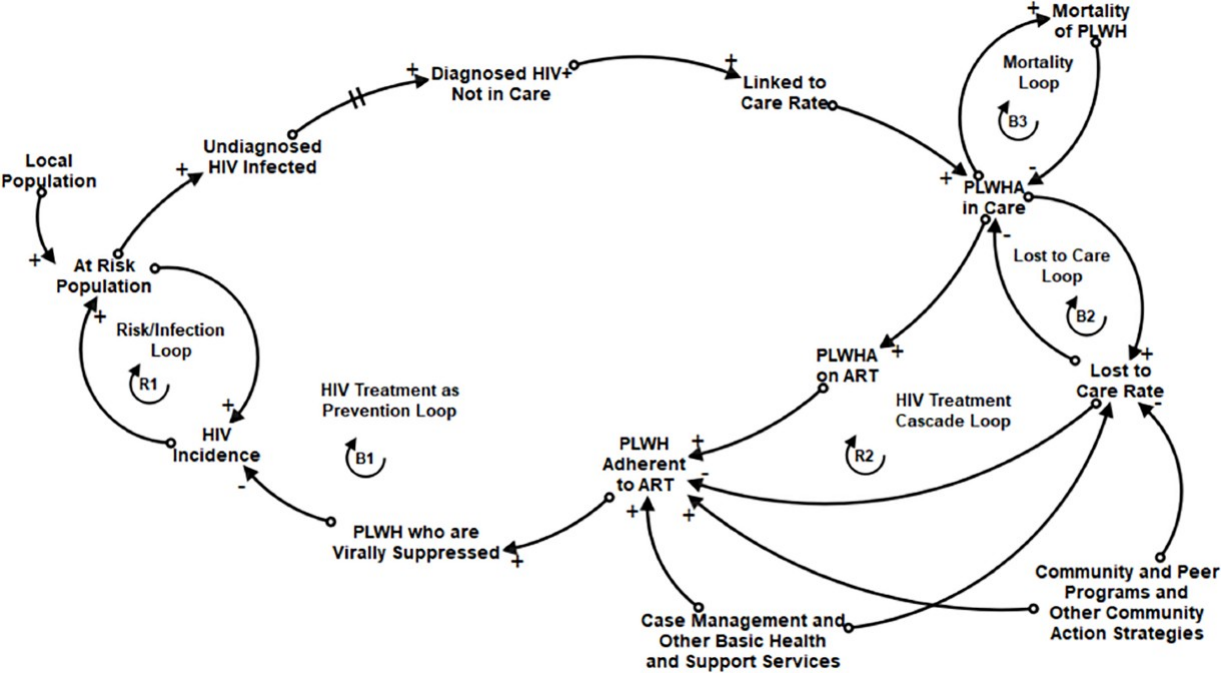
Causal loop diagram: HIV treatment as prevention. This CLD shows two reinforcing feedback structures (R1 and R2) and three balancing feedback structures (B1, B2 and B3) that collectively represent HIV burden in the community, in relation to basic services and action strategies that serve to foster access to HIV care, use of antiretroviral therapy (ART), and HIV testing. Positively associated connections (+) indicate variables that change in the same direction as each other; negatively associated connections (-) indicate variables that change in the opposite direction as each other.

Module A reflects the main structure of the HIV CC model. It represents the significance of delays and feedback loops in the HIV CC system that could diminish or improve community-level health outcomes over time. The conceptual design of this structure, which reflects TasP [4, 8], is represented by the CLD in **Fig 2**.

This SD conceptual model comprises five feedback loops evident in the CLD. The “**Risk/Infection Loop**” **(R1)** is a reinforcing feedback loop representing the rate of infection of people in the local population who are at risk. As more of the at-risk population is infected but has not yet achieved viral suppression, HIV incidence rises. This, in turn, increases the likelihood of people being at risk of exposure and infection. This reinforcing feedback loop can only be slowed by reducing HIV incidence through prevention efforts, including TasP. The “**HIV Treatment as Prevention Loop**” **(B1)** is a balancing feedback loop. In this loop, undiagnosed HIV infected people become diagnosed, linked to care, in medical care, on ART, and adherent to ART, thereby achieving viral suppression, resulting in lower HIV incidence [4, 56, 64]. The reinforcing “**HIV Treatment Cascade Loop**” **(R2)** begins with HIV incidence and undiagnosed PLWH being diagnosed and linked to care, but then becoming lost to care and non-adherent to medication. This likely results in high “community viral load” [54, 55] and high potential for increased HIV incidence. Preventing this treatment cascade is the goal of the whole HIV CC system and community efforts to support and care for PLWH. The balancing “**Lost to Care Loop**” **(B2)** reduces the total number of PLWH in the care system, as does the balancing “**Mortality Loop,**” **(B3)** representing the death rate of PLWH.

The CLD additionally depicts two kinds of community resources outside direct HIV medical care services that contribute to system outcomes. These include Basic Services, such as Ryan White medical case management, housing, substance use treatment, and mental health services. These community resources are expected to increase the rate of PLWH being linked to medical care and the number who achieve and maintain viral suppression by keeping them on their medications and preventing them from becoming lost to care. In addition, many communities conduct evidence-based and other community interventions to supplement and improve the HIV CC system. These may include community and peer programing designed to support PLWH and their families by increasing HIV awareness and reducing stigma and medical mistrust at the community level. These hypothetical Action Strategies are expected to keep PLWH on their medication and reduce the number lost to care, as well as increase overall community support for and improve wellness of PLWH and their families.

### Stock-and-flow diagram

**Fig 3:**
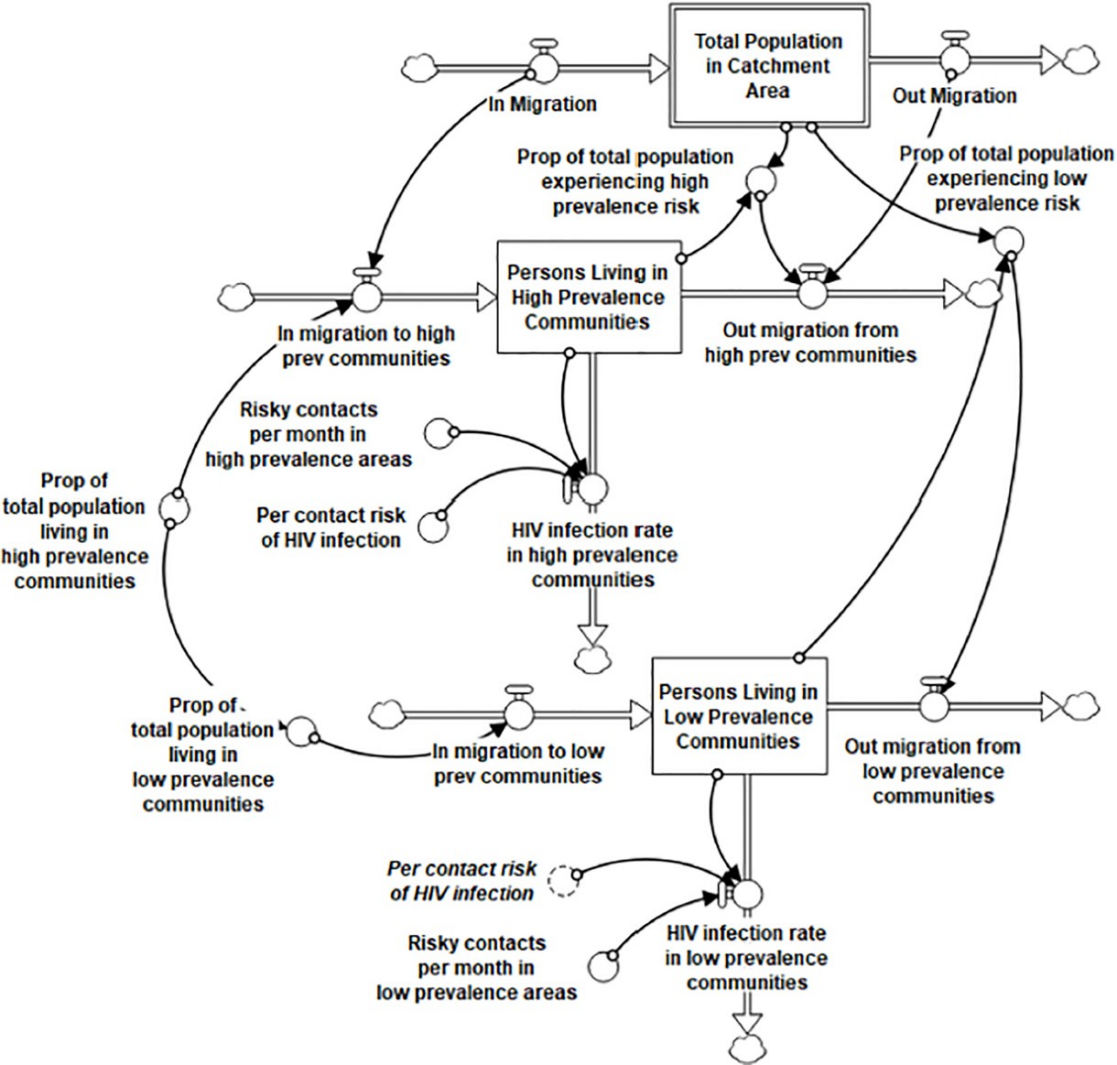
Stock-and-flow diagram: Population and HIV incidence (Module A, partial). Depicts total population in the catchment area, disaggregated into ‘high’ and ‘low’ HIV prevalence communities, and the factors driving HIV infection, or incidence, over time.

**Fig 4:**
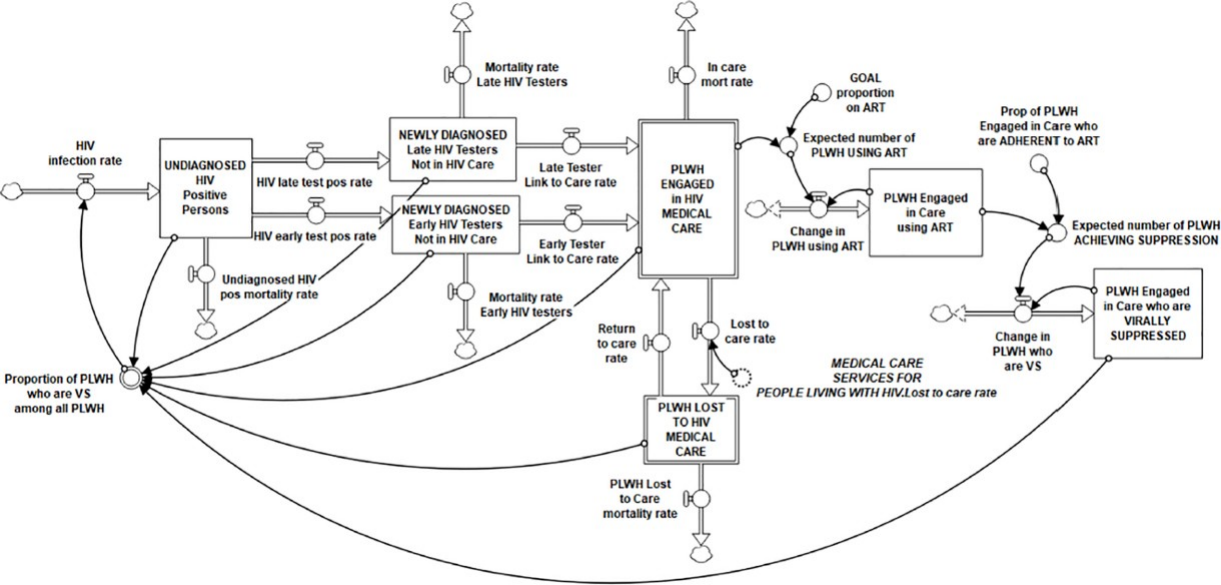
Stock-and-flow diagram: HIV treatment cascade (Module A, partial). Newly infected persons transition through the treatment cascade over time, moving from being UNDIAGNOSED, to DIAGNOSED, to ENGAGED IN CARE, to achieving VIRAL SUPPRESSION.

Two linked computational stock and flow components of the HIV Infection and Treatment as Prevention Module are shown in **Figs 3 and 4**. In these diagrams, stocks are represented by boxes, and flows are represented by uni-directional or bi-directional pipes with valves symbolizing regulators of speed or volume into or out of the stock. Converters, or auxiliary variables, (circles) are linked to stocks, flows, and each other (indicated by directional arrows) in underlying equations that generate the inter-relational system dynamics over time, such as rates of flow, delays, and accelerators or decelerators that contribute to dynamic system outputs.

Every stock has an initial value based on data or information used to define that variable at a specific starting time point for the catchment area. All model parameters are held constant throughout the simulated time horizon. Each flow in the model is calibrated with a formula that factors in either a time variable (e.g., number of persons per month) or a proportion variable (e.g., proportion of PLWH in a particular state who move to the another state), or both, and may also include other factors assumed to affect those rates of change.

The full set of initial parameters and formulas for Module A is included in the **S1 Table** in the supplemental materials of this article. Some factors have been imported into this module from other modules in the full HIV CC SD model listed in Table 1, where they are being generated by the complex dynamics in those subsystems. These and all variables in the stock and flow models in each of the nine modules are available in similar tables in our online repository (https://github.com/mweeks56/ICR_HIV_Care_SDM) and on the project’s protocols website at dx.doi.org/10.17504/protocols.io.bcm6iu9e.

To begin the flow of people through the HIV Infection and Treatment as Prevention module, the Population and HIV Incidence subsection (Fig 3) generates the HIV incidence rate from the total population in the catchment area. Because of the greater likelihood of having a transmission encounter where HIV is more prevalent, we divided the total population into those living in a locale with high HIV prevalence (defined as >500 PLWH per 10,000 residents) and those living in low HIV prevalence areas. In our catchment area, two cities met the criterion of high prevalence (16.2% of the total population in the catchment area), and the remainder of the population lives in low prevalence areas. These two stocks have the same in-/out-migration rates as the total population for the purpose of our base case scenario.

An additional outflow from these stocks calibrates the monthly rate of infection, which is driven by three key variables in the model: the “risky contacts per month” in high and low prevalence areas, the “per contact risk of HIV infection,” and the “proportion of PLWH who are VS [virally suppressed] among all PLWH.” Risky contacts per month is set at .009 in high prevalence areas and .003 in low prevalence areas (per 10,000 risky encounters, in a ratio of 3:1 high: low; risky contacts in high prevalence areas is discussed in more detail below.) Per contact risk of infection is set at .0049 in both types of settings, calibrated as the mean likelihood of infection from any kind of sexual and injection risk, as developed by the CDC [65]. The proportion of all PLWH who are VS is a dynamic variable generated by model simulation, formulated as diagnosed PLWH known to be virally suppressed in the numerator (initial value in the catchment area is 0.65 of diagnosed PLWH by 2017 [59], see S1 Table) and the total number of diagnosed plus estimated undiagnosed PLWH as the denominator. Taken together, the HIV infection rates in high plus low prevalence areas generate the HIV infection rate for the total model (see Fig 4).

The flows in Fig 4, the HIV Treatment Cascade subsection of Module A, represent the number of PLWH per month who transfer from one status along the cascade to another. The HIV infection rate, generated in the Population and HIV Incidence subsection (Fig 3), begins the flow of PLWH into the treatment cascade. HIV incidence in our base case scenario was calibrated to produce the number of new cases in the catchment area documented in state surveillance, with the addition of the estimated rate of undiagnosed infected. To estimate undiagnosed PLWH, the CT DPH uses a procedure adopted by the U.S. CDC based on surveillance data and CD4 counts [66, 67]. By the end of 2016 in CT, the annual estimated diagnosed PLWH was 90.2% (CI 77.9–100%), and preliminary data through 2018 indicated 90.7% (CI 78.8–100%) [67]. Therefore, we applied the state estimate of 10% undiagnosed to produce the total number of new infections for our catchment area (see S1 Table).

Incidence feeds into the stock of undiagnosed PLWH, who are then separated into two important outflows, the HIV late test positive rate and the HIV early test positive rate. Late testing is defined as having received an AIDS diagnosis concurrent with or within 12 months of an HIV diagnosis, reported as 22% of newly diagnosed in CT in 2017 [46]. Late testing is a significant problem for the HIV CC because of the high likelihood that late testers will unwittingly spread the virus over a longer period of time and because their burden of disease and mortality outcomes are significantly worse than for early testers [68]. We used the “ghosting” tool in Stella Architect®, whereby a variable in one module can be brought into a formulation in another, in order to import the HIV testing rate in the catchment area from Module B, HIV Prevention and Testing. This rate was approximately 8.1 persons per month, or 97–100 new diagnoses per year matching the 2012–2017 average number of annual new infections in the area (range: 73–120, M = 97.33/year).

Linking diagnosed PLWH to their initial medical appointment is a significant transition in the HIV care continuum, and one subject to substantial delays in the past. However, new CDC “test and treat” protocols to start medication for newly diagnosed PLWH immediately [69, 70], coupled with U.S. HRSA-funded social supports such as Ryan White medical case management, have dramatically reduced wait time between HIV diagnosis and an initial medical visit in Connecticut and many other states over the past two decades. Case management services have long been demonstrated to reduce time between HIV diagnosis and linkage to care and facilitate retention in care [63, 71]. The linked to care rate (defined by the CDC as having had an initial CD4 and VL count reported to the state DPH), is generated by dynamics produced in Module C, Medical Care Services, and imported into this module. This linked to care rate was applied to both early and late testers, which were divided according to the proportion of late testers in the catchment area. These in-flows feed the stock of PLWH Engaged in HIV Medical Care, the primary goal of the care continuum needed to achieve viral suppression in all infected persons. PLWH who pass through all earlier stages of the treatment cascade cannot return to a previous stage, though they might remain in any one stage (stock) for a significant period of time. Death (mortality rate) is the only way PLWH are permanently removed after having been engaged in care. The number in the Engaged in Care stock is generally expected to rise for some time to come, with more newly infected coming in and efficacious ART treatment significantly extending the lifespan of infected persons, thereby slowing the outflow through mortality.

A portion of those engaged in care are also represented in the stock of PLWH using ART, the inflow into which is determined by the expected number of PLWH using ART multiplied by the time it takes to start ART. Further, a proportion of the PLWH Engaged in Care who are using ART is represented in the stock of virally suppressed (VS) PLWH. The rate of flow into that stock is determined by the estimated proportion who are adherent to their medication regimen and the time it takes to become VS after starting ART (see S1 Table for all these parameters and formulas).

However, despite initial engagement in care, many PLWH do not achieve or cannot maintain VS. Protocols for when to initiate ART and protocol implementation continue to vary despite CDC recommendations to begin treatment immediately upon diagnosis, as well as increasingly popular state and community campaigns to promote TasP (e.g., U = U: Undetectable = Untransmittable) [72] and setting the goal of treating 100% of PLWH. Further, patients face many challenges and limitations to lifelong consistent adherence to ART [73]. Bidirectional flows represent net change of PLWH using ART and PLWH who are VS. Significant federal, state and local resources are brought to bear on keeping patients on their medication and attending medical appointments to achieve and sustain VS. This component of the HIV CC illustrates the fundamental assumption of TasP, i.e., that medical engagement and treatment adherence contribute to the goal of achieving VS in all PLWH, which is expected to reduce new HIV incidence at the community level.

The biggest challenge to achieving the benefit of the HIV care continuum on TasP is the problem of patients being lost to HIV medical care and therefore assumed to be non-adherent to ART and likely to increase community viral load. The two opposing flows between the stocks of Engaged in Care and Lost to Care represent the potential for someone who has been linked to care initially to move back and forth between these two states. The lost to care rate that feeds the stock of lost to care patients, the rate of their return to care, and the dynamics associated with these processes, are imported here from Module C, Medical Care Services. There, they are driven by several factors, including the “Effect of unmet service needs on risk of being lost to care” (imported from the four Basic Services Modules B-E) and the “Effect of action strategies on the risk of being lost to care” (imported from Module G, Peer Advocacy, and Module I, Mobilizing Community Initiatives). (Details on these inputs from each of these modules can be found on our on-line repository and protocol websites indicated above.) Taken together, these positive and negative dynamics have significant impact on the success of the HIV CC to achieve viral suppression in all PLWH by keeping them in medical care over time.

### TasP dynamics

As shown in the stock-and-flow diagram (Fig 4), the primary HIV TasP balancing feedback loop (B1 in Fig 2) can be seen in the flow of the newly infected (HIV incidence rate) into the stock of undiagnosed PLWH, through early or late diagnosis and linkage to care into the stock of PLWH engaged in HIV medical care and viral suppression. Success of the system contributes to a higher proportion of VS PLWH in the community and ultimately a reduction in the HIV incidence rate. We represent the proportion of VS PLWH in two ways. One is described above, in which PLWH in the Virally Suppressed stock provides the numerator and all stocks of diagnosed and undiagnosed PLWH along the care continuum form the denominator, the full circle of the balancing HIV CC feedback loop. We also represented the proportion VS of those diagnosed with HIV (absent the undiagnosed), which is the figure reported by state departments of health, to assist with validation of our model in comparison to known trends in VS.

Also evident is the reinforcing treatment cascade feedback loop (R2 in Fig 2). This is the result of the “vicious cycle” of being lost to care on ART adherence and the number of PLWH who are VS. This contributes to an overall reduction in the proportion VS among all infected persons, and potentially to increased HIV incidence.

## Pattern tests: Comparison of reported to simulated reference modes for key variables

The computational model structure allows for user specification of key epidemiological parameters and other conditional variables in the model to reflect local conditions at the start of the intended time horizon. As indicated above, our SD model parameters and equations were calibrated to simulate the base case scenario over a time horizon of 60 months (5 years; corresponding to calendar months t_0_ = 01/01/2018 and t_60_ = 01/01/2023). The prior calendar year (2017; t_-12_ = 01/01/2017 to t_-1_ = 12/31/2017) was used to confirm model production of local HIV CC outcomes by comparing base case simulated output with reported data trends over the same period. As indicated above, the prior year was also used to resolve any computational anomalies due to initial parameter values ahead of T_0_. All initial parameter values were derived from publicly available demographics and CT HIV surveillance data for 2015–2017 [46].

**Fig 5:**
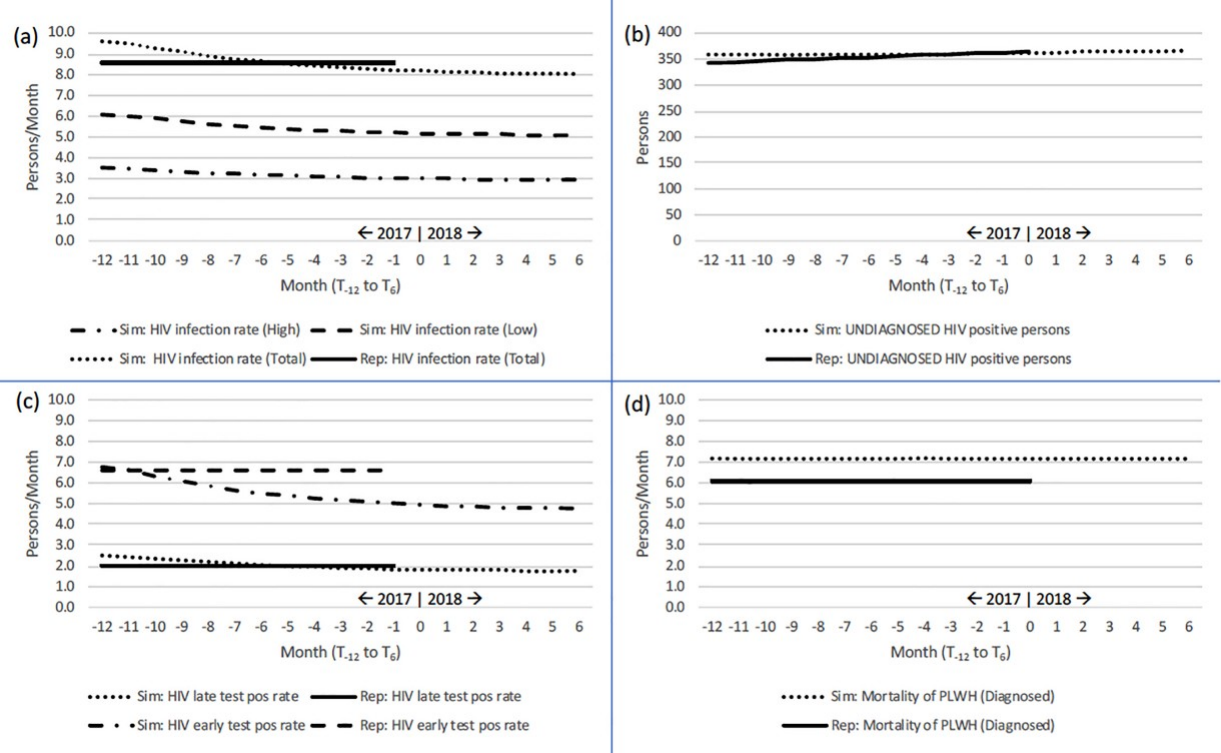
Pattern tests: Comparison of reported to simulated reference modes, 1-Jan-2017 to 1-Jul-2018 (T_-12_ to T_6_). (a) HIV infection rate; (b) Undiagnosed HIV positive persons; (c) HIV test positive rate; and (d) HIV-specific mortality.

**Fig 5** presents line graphs in four panels for: (a) HIV infection rate, (b) Undiagnosed HIV positive persons, (c) HIV test positive rates, and (d) HIV-related mortality. By observation, simulated HIV infection rates at T_-1_ differ from reported HIV incidence by 0.7 persons/month, with simulated output at 9.3 persons/month and reported data at 8.6 persons/month. The flat pattern and minor difference in magnitude suggest that the model performs well for incidence, with the slightly higher simulated output reflecting inclusion of those persons who are living with HIV but who have yet to be diagnosed. Applying CT reports indicating that estimates of the number of undiagnosed persons is approximately 10% of all know PLWH, our model is well calibrated, showing no difference in pattern or magnitude for this category. Likewise, simulated data for both early and late HIV positive test rates is negligibly dissimilar. Notably, our model over simulated HIV mortality relative to reported data (+3 persons/month). This over-estimate may reflect that our model includes deaths among persons still undiagnosed whose cause of death may have been misclassified.

**Fig 6:**
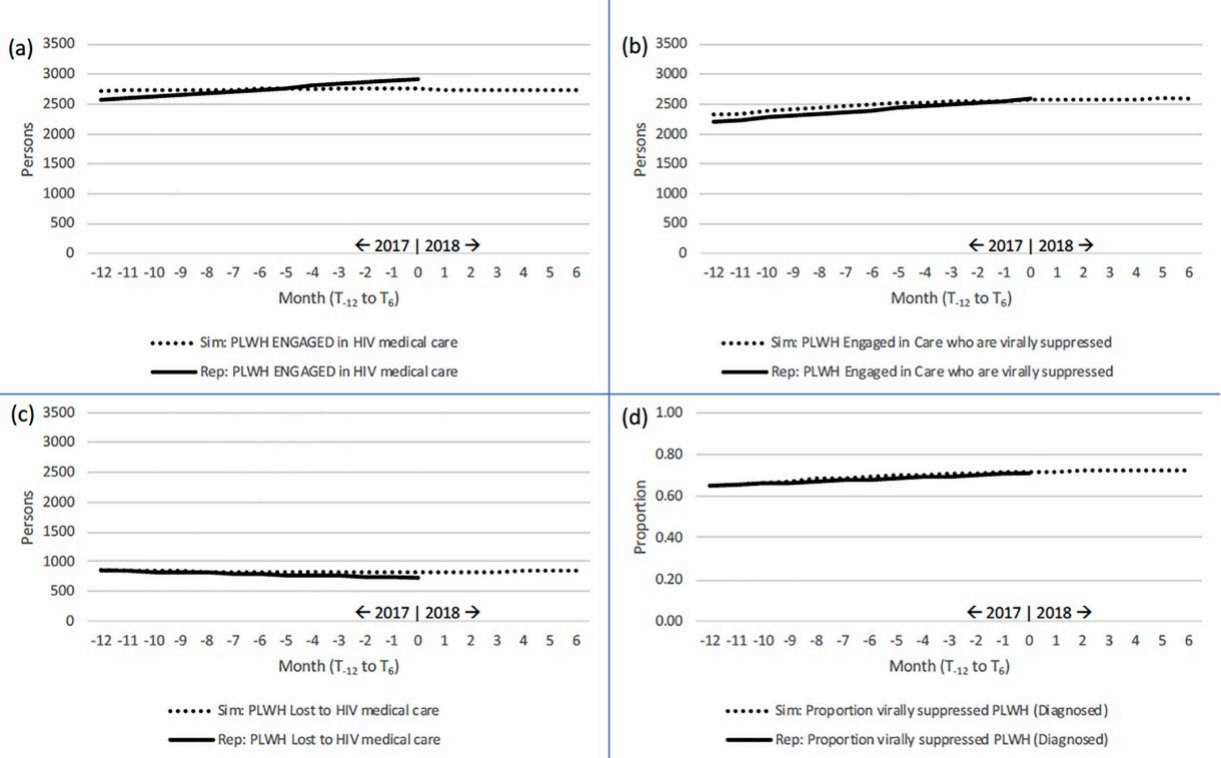
Pattern tests: Comparison of reported to simulated reference modes, 1-Jan-2017 to 1-Jul-2018 (T_-12_ to T_6_). (a) Engaged in care; (b) Engaged in care and virally suppressed; (c) Lost to care; and (d) Proportion virally suppressed.

**Fig 6** presents line graphs in four additional panels for: (a) PLWH engaged in HIV medical care, (b) PLWH engaged and virally suppressed, (c) PLWH lost to HIV medical care, and (d) the proportion of PLWH who are virally suppressed (among diagnosed only). Again by observation, we see that simulated to reported data trends are negligibly different in pattern and magnitude for all featured variables.

### Sensitivity analysis: Proportion of PLWH who are virally suppressed

To further validate model behavior associated with the effect of TasP, we conducted a sensitivity analysis comprising three key parameters for which there is limited evidence, namely: (1) Risky Contacts per Month in High Prevalence Communities, (2) the Proportion of PLWH Engaged in Care who are adherent to ART, and (3) the Initial Proportion of No Shows Lost to Care (see **Table 2**). The first variable is in the Population and HIV Incidence segment of Module A (Fig 3), the second is in the HIV Treatment Cascade segment of Module A (Fig 4), and the third is in Module C, Medical Care Services (not shown).

**Table 2:** Parameters selected for sensitivity analysis of proportion of PLWH who are virally suppressed.

Parameter	Lower	Base case estimate	Upper
Risky contacts per month in high prevalence areas	.008	.009	.010
Prop of PLWH Engaged in Care who are ADHERENT to ART	.850	.950	1.0
Initial Prop of No Shows Lost to Care	.500	.600	.700

Our base case value for “Risky Contacts per Month in High Prevalence Areas” (.009 persons/person/month) is the rate of HIV exposure among residents living in a high HIV prevalence community within our targeted catchment area. The base case estimate was determined via model calibration to reflect known historical HIV diagnosis rates for the total catchment area (8.6 persons/month) and known difference in diagnosed cases of HIV in high to low prevalence communities (we used a 3:1 ratio for high: low HIV prevalence communities). Our base case value for the “Proportion of PLWH Engaged in Care who are Adherent to ART” (95%) is the expected proportion of virally suppressed PLWH out of all PLWH who were engaged in care in the catchment area (Hartford TGA) in 2017. This estimate is reported by the CT DPH [59]. Finally, the base case value for the “Initial Proportion of No Shows Lost to Care” was estimated to be 60% by our Task Force members. This stakeholder-estimated proportion applies HRSA’s definition of lost to care (not having seen a medical provider within a 12-month reporting period) as well as local medical provider protocols and clinical experiences (frequency of missed appointments of PLWH patients who have been scheduled for 6-month or 12-month clinical visits).

We selected upper and lower bounds for each parameter included in the sensitivity analysis around our base case estimate (see Table 2). The upper and lower bounds for “Risky contacts per month in high prevalence areas” (contacts/person/month) was set to +/- 10% of the base case value. For the “Proportion of PLWH Engaged in Care who are ADHERENT to ART,” the upper bound was set to 100% and the lower bound was set to -10% of the base case value (85%). Similarly, for “Initial Proportion of No Shows Lost to Care,” upper and lower bounds were set to +/- 10% of the base case value. Decisions regarding the range for each parameter were based upon available reports and opinion of participating public health experts.

**Fig 7:**
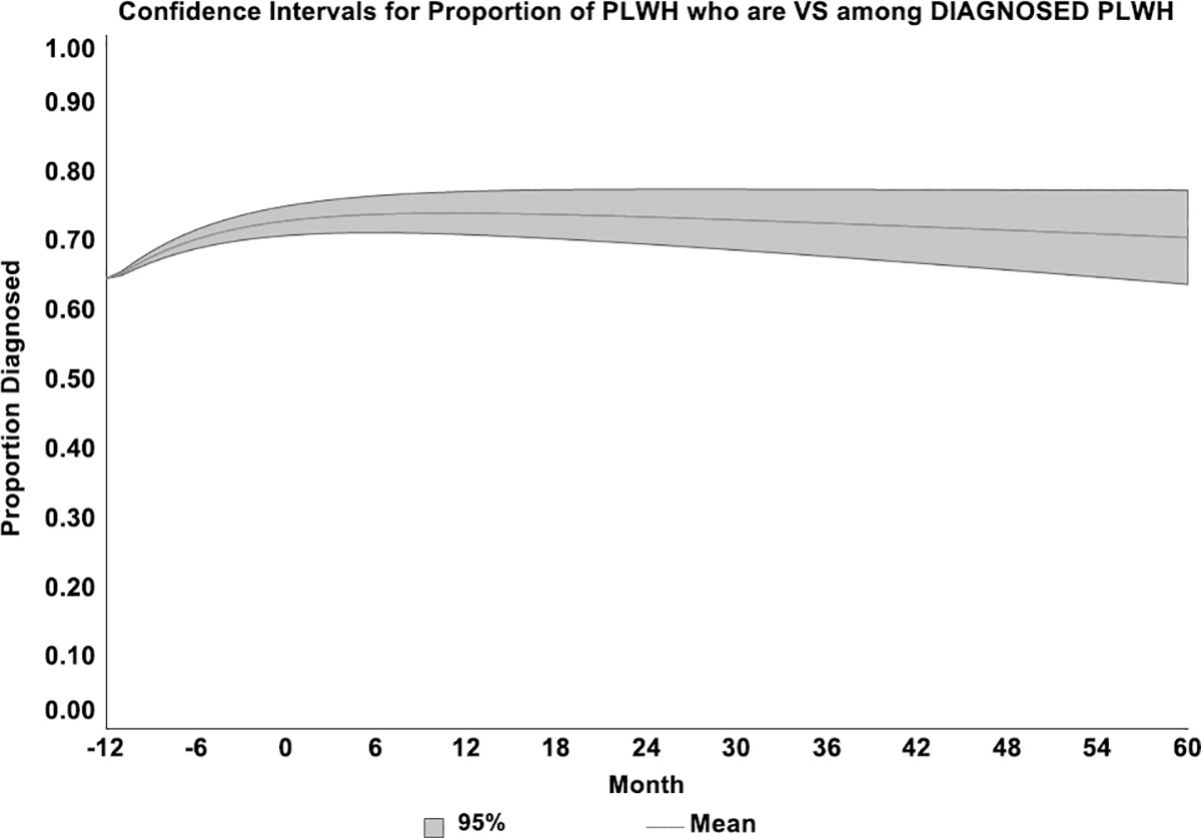
Sensitivity analysis. 95% Confidence Interval about the Proportion of PLWH who are Virally Suppressed (VS) among Diagnosed PLWH. Three parameters: (1) Risky contacts per month in high prevalence areas (range: .008 –.010; base case value = .009); (2) Prop of PLWH Engaged in Care who are ADHERENT to ART (range: .85–1.0; base case value = .95); and (3) Initial Proportion of No Shows Lost to Care (range: .50 –.70; base case value = .60). Sample runs: 3 parameters x 3 values x 3 combinations = 27.

**Fig 7** displays a 95% confidence interval based upon N = 27 sample runs (3 parameters x 3 values [low, base case, high] x 3 combinations per value) for the variable “Proportion of PLWH who are Virally Suppressed (VS) among Diagnosed PLWH.” Results indicate that simulated outcomes are relatively stable across this set of sample runs, with base case values ranging between 63.7% and 78.2% (Mean = 70.9%) by Month T_60_.

## Discussion and model applications

A growing body of literature affirms the benefits of using participatory SD modeling and GMB in partnership with relevant stakeholders to examine and seek strategies to address complex problems like the HIV epidemic [32, 74–76]. As described above, we followed best practice protocols widely acknowledged by the SD modeling community for iterative model development and validation in partnership with our community stakeholder SD Modeling Task Force [45, 48, 51]. Our GMB sessions were designed to systematically build segments of the model structure, based on group narrative of the HIV CC system and healthcare and service delivery processes, and to critique that model structure as it was being built [39]. As Task Force members gained more SD modeling knowledge and insight over time, their input into model conceptualization improved greatly. GMB scripts solicited their input and critiques of draft models and the need for and utility of adding or removing specific variables, defining new feedback structures, or checking equation dimensions and parameter estimates. Reliability of model behavior was tested through repeated simulations of the base case scenario until it approximated current epidemiological trends in the catchment area and other system dynamics that resonated with participants and their experiences with the HIV care and support system.

An important aspect of model construct validity hinges on acceptance of underlying assumptions about TasP, which is an increasingly accepted framework, both in the public health literature and in communities, for understanding and improving community level efforts to control the HIV epidemic [77]. The HIV CC SD model is designed to include all key constructs relevant to the concept of TasP, including those that define the basic care continuum and treatment cascade, as well as typically available basic services for HIV testing, medical care, case management, and supportive housing, substance use treatment, and mental health care. It also includes Task Force identified and commonly available community level interventions designed as optional action strategies, which can be left inactive or turned on to simulate the effects of these types of programs when implemented in the local area.

Data validity was assured by relying heavily on DPH surveillance reports and available data on Ryan White and other resources that support PLWH in the catchment area, as well as our own primary data of local HIV care service utilization, time delays, and experiences of stigma from our study’s cohort participants. A series of tests of our base case scenario against known epidemiological and service utilization trends and patterns provided confidence in our model structure and behavior. Variables with no known data were deliberated to seek a method of calibration that was consistent with Task Force members’ real world experiences. Selected parameters with greater uncertainty were used in sensitivity analyses; these analyses indicated that the model performed well for a wide range of plausible values for the catchment area. This comprehensive approach to model data validation helped our research team and the community SD Modeling Task Force to feel confident in the content, structure, parameters, and outputs of the model.

The significance and value of a validated SD model hinges on its usability and usefulness by community stakeholder groups. In general, SD models can be used for three main purposes: 1) for stakeholders to learn about system complexity; 2) to identify drivers of system dynamics and leverage points expected to generate desired system change; and 3) to test strategies and seek optimal resource allocations through simulation in order to deliberate priorities and preferences for improving system outcomes.

The construction and validation of the HIV CC SD model makes it possible for stakeholders to propose, run simulations of, and test potential impacts of various hypothetical scenarios representing intervention strategies and other leverage expected to increase viral suppression in PLWH and reduce HIV incidence at the population level. An unlimited number of scenarios can be hypothesized and simulated. Some could include modifying parameters of the basic epidemiological variables, revising time estimates of moving PLWH through stages of the HIV care continuum, or changing other proportional variables set by the community (e.g., those listed in S1 Table in the appendix and other variable tables on the project’s online repository and protocol websites indicated above). These allow tailoring the base case scenario to later time points in the same catchment area or local conditions in other communities. Other simulations are done for the purpose of generating “what if” scenarios [27, 32, 78, 79] to examine the effects of changes expected to achieve system improvements. These could include programmatic changes, such as increasing available human resources for provision of basic services, like case managers, early intervention specialists, or medical providers, in the basic services modules. Or they could include initiating (“turning on”) or strengthening peer and community programs and other hypothetical “action strategies” to increase support for PLWH and reverse the impacts of stigma and other negative community norms, among other possibilities. “What if” scenarios can be initiated at the start of the run, part way into the run, concurrently, or sequentially to examine impacts on key health indicators or test various strategies or combinations anticipated to improve health outcomes. Decisions on which scenarios to simulate are best achieved through deliberation by a stakeholder group, whose common goal it is to improve system functioning [80, 81].

With the option of locally tailoring key modifiable variables in the model, the whole HIV CC SD model is designed to be used in other communities besides the one in which it was developed. Further, our systematic comparison of simulated output with reported trend data on key HIV CC metrics provides a viable method to assess the degree to which model tailoring can improve applicability and generalizability of the whole model structure to other communities. The primary benefit of the model is to foster deeper understanding about the feedback that drives complex community-based HIV care delivery in any community.

Nevertheless, generalizability of the model has several limitations. First, confirmation of the model’s behavioral validity is limited until it can be tested in a setting other than the one in which it was originally calibrated. Additionally, this model focuses on the HIV CC service system at the community and total population level. Many communities and potential model users are interested in specific experiences of subpopulations with disproportionate risk or rates of infection and other disparities. Though it may be possible to calibrate the model to examine one or another specific subpopulation, it is not intended for that purpose, but rather to illustrate and simulate the dynamics of the whole community’s effectiveness in achieving TasP and overall care for PLWH in that community. Further, as indicated above, some variables have no known metric, and are informed by best estimates provided by the literature or community experiences and perceptions. Our initial tests of the model through comparison of the DPH documented HIV epidemiological trends with our simulated base case scenario and the sensitivity analyses we conducted have helped to affirm that many of these “soft” variables (e.g., risky contacts per month in high/low prevalence areas, proportion of PLWH adherent to ART, proportion of no-shows who are lost to medical care, etc.) are within reasonable approximation of real world conditions in the local setting. Finally, simulation runs can forecast likely trends and changes in direction of key system variables, but the number of interacting factors and potential for exogenous factors to impact system dynamics suggests caution for predicting future system changes.

Model application is often limited by the technical complicatedness of its use and complexity or ambiguity of modeling results. User friendly tools, such as apps, dashboards, and other model interfaces designed to facilitate layperson use of the simulation model, are often needed to make SD models available to publics that can benefit from their use. At the time of this writing, we were in the initial phase of developing a user interface with features built into Stella Architect® to help streamline public use of our HIV CC SD simulation model. We were also testing application of the model in collaboration with a regional planning council to assist them with their priority-setting and strategic planning needs. We anticipate learning more about the process, benefits, and limitations of using this model in a community group planning process and its potential applications for others.

Our experiences with building, validating, and testing the use of this SD simulation model of the HIV CC service system has demonstrated the importance of engaging diverse stakeholders in a process of visualizing, critiquing, and seeking ways to comprehend the vast complexity of a healthcare delivery system. Such broad and deep vision is necessary to identify game changing actions to achieve the elimination of an epidemic like HIV.

## Supporting information

S1 Table HIV care continuum system dynamics model variables, definitions, and calibrations: HIV infection and treatment as prevention module. (DOCX)
