# Supplementary material for: Simulating system dynamics of the HIV care continuum to achieve treatment as prevention
Source: PLoS One. 2020 Mar 19;15(3):e0230568. doi: 10.1371/journal.pone.0230568 (PMC7082036; doi:10.1371/journal.pone.0230568)
Supplement: S1 Table — (DOCX) [file pone.0230568.s001.docx]

**S1 Table. HIV Care Continuum System Dynamics Model Variables, Definitions, and Calibrations: HIV Infection and Treatment as Prevention Module**

| **Variable Name** | **Variable Type** | **Unit** | **Initial Parameter Values and Formulas** | | **Variable Definition/Specification and**  **Sources of Initial Parameters and Stock Values** |
| --- | --- | --- | --- | --- | --- |
| **Initial Values of the HIV Care Continuum and Population** | | | | | |
| Initial total population in catchment area | auxiliary | Persons | 1,210,256 | | Census estimated 2017 population in the HRSA designated Ryan White Hartford, TGA (Transitional Grant Area), which includes Hartford County (895,388) + Tolland County (151,458) + Middlesex County (163,410), Connecticut. |
| State DPH estimate of UNDIAGNOSED PLWH | auxiliary | dmnl | 0.10 | | Estimated proportion of undiagnosed people living with HIV (PLWH) in CT. Calibrated by CT Dept. of Public Health (DPH) with the CDC-approved method of using CD4 data to estimate undiagnosed HIV infections.^1,2^ |
| Init UNDIAGNOSED | auxiliary | Persons | Init DIAGNOSED * State DPH estimate of UNDIAGNOSED PLWH | | Initial estimated number of undiagnosed PLWH in the catchment area, formulated with the 2017 number of diagnosed PLWH reported to CT DPH HIV Surveillance Division multiplied by the CT DPH estimated proportion of undiagnosed PLWH in 2017.^1,2^ |
| Init DIAGNOSED PLWH | auxiliary | Persons | 3586 | | Number of people diagnosed with HIV living in the catchment area (Hartford TGA) as reported to CT DPH by 12/31/2016. HIV Surveillance data through December 2017.^3,4^ |
| Init TOTAL PLWH | auxiliary | Persons | Init UNDIAGNOSED + Init DIAGNOSED | | Formulated using the number of people diagnosed with HIV in the Hartford TGA reported to CT DPH by the end of 2017^4^ plus the 2017 calibrated number of undiagnosed PLWH in CT.^1,2^ |
| Init prop ENGAGED IN CARE | auxiliary | dmnl | 0.76 | | Proportion of diagnosed PLWH engaged in HIV care (>=1 visit in prior 12 month reporting period) of all diagnosed PLWH in the Hartford TGA by 12/31/2016. CT DPH HIV Surveillance Div.: HIV Continuum of Care in the Hartford TGA, 2017.^4^ |
| Init prop of DIAGNOSED PLWH LOST TO CARE | auxiliary | Persons | 0.24 | | Proportion of PLWH who were lost to care in Hartford TGA by 12/31/2016, defined as no CD4/VL test results reported to CT DPH in the prior 12 months reporting period. CT DPH Continuum of Care, 2017.^4^ |
| Init est prop of Diagnosed on ART | auxiliary | dmnl | 0.76 | | Estimated proportion of PLWH on antiretroviral treatment (ART). Calibrated to equal the proportion of PLWH engaged in care in Hartford TGA by 12/31/2016, CT DPH, HIV Continuum of Care^4^ |
| Init prop VIRALLY SUPPRESSED | auxiliary | Persons | 0.65 | | Proportion of diagnosed PLWH who were virally suppressed in the Hartford TGA by 12/31/2016, defined as ≤ 200 copies/ml. CT DPH, CT HIV Continuum of Care, 2017^4^ |
| Initial documented HIV incidence (diagnosis) rate for catchment area | auxiliary | persons/ month | 8.25 | | Average monthly rate of new unduplicated HIV diagnoses from all testing sites in Hartford TGA, reported to CT DPH in response to mandatory reporting regulations. Calibration based on annual test rate of approximately 99 newly diagnosed PLWH/year for the years 2015-2018. CT DPH HIV Surveillance, 2017.^3,4^ |
| **Variable Name** | **Variable Type** | **Unit** | **Initial Parameter Values and Formulas** | | **Variable Definition/Specification and**  **Sources of Initial Parameters and Stock Values** |
| **Population Distribution and HIV Infection Dynamics** | | | | | |
| Total Population in Catchment Area | stock | Persons | | Total Population in Catchment Area(t - dt) + (In Migration - Out Migration) * dt {NON-NEGATIVE}  Initial value = Initial total population in catchment area | Number of people living in the Hartford TGA over time. |
| In migration rate | auxiliary | 1/month | | .01 | Estimated rate of population migration into the catchment area. This parameter is equal to outmigration to hold population change steady in simulation runs. This is generally reflective of the Hartford TGA in the past 5 years. |
| Out migration rate | auxiliary | 1/month | | .01 | Estimated rate of population migration out of the catchment area. This parameter is equal to in-migration to hold population change steady in simulation runs. |
| In Migration | flow | Persons/month | | Total Population in Catchment Area * In migration rate {UNIFLOW} | Monthly rate of change in migration into the catchment area. |
| Out Migration | flow | Persons/month | | Total Population in Catchment Area * Out migration rate {UNIFLOW} | Monthly rate of change in migration out of the catchment area. |
| Prop of total population living in high prevalence communities | auxiliary | dmnl | | .162 | Proportion of people living in communities in the catchment area (Hartford TGA) with highest HIV prevalence in 2017, defined as >500/10K residents (including cities of Hartford and New Britain) over the total population in the Hartford TGA. CT DPH HIV Surveillance Map of HIV in CT. |
| In migration to high prev communities | flow | Person/ Month | | In Migration * Prop of total population living in high prevalence communities {UNIFLOW} | Monthly rate of migration into the high HIV prevalence communities, applying the same in-migration rate as to the whole catchment area. |
| Persons Living in High Prevalence Communities | stock | Persons | | Persons Living in High Prevalence Communities(t - dt) + (In migration to high prev communities - Out migration from high prev communities - HIV infection rate in high prevalence communities) * dt {NON-NEGATIVE  Initial value = Initial total population in catchment area * Prop of total population living in high prevalence communities | Number of people in the Hartford TGA living in communities with highest prevalence rates in 2017 (>500/10K residents, including cities of Hartford and New Britain) over time. |
| Prop of total population experiencing high prevalence risk | auxiliary | dmnl | | Persons Living in High Prevalence Communities / Total Population in Catchment Area | Proportion of residents in the catchment area living in high HIV prevalence communities over time in simulations. |
| Out migration from high prev communities | flow | Person/ Month | | Prop of total population experiencing high prevalence risk * Out Migration {UNIFLOW} | Monthly rate of migration out of the high HIV prevalence communities, applying the same out-migration rate as to the whole catchment area. |
| Risky contacts per month in high prevalence areas | auxiliary | Contacts/Person/ Month | | .009 | Estimated rate of HIV exposure per month of residents living in a high HIV prevalence community within the targeted catchment area (Hartford TGA). Determined via model calibration to reflect known historical HIV diagnosis rates for the total catchment area (8.25 persons/month) and the known difference in diagnosed cases of HIV in high to low prevalence communities (3:1 ratio). Note: Affirming uncertainty in the true value of this parameter, sensitivity to changes in the “proportion of virally suppressed of diagnosed PLWH” relative to this risky contact parameter was assessed (Range = .0085 +/- 10%). |
| **Variable Name** | **Variable Type** | **Unit** | **Initial Parameter Values and Formulas** | | **Variable Definition/Specification and**  **Sources of Initial Parameters and Stock Values** |
| Per contact risk of HIV infection | auxiliary | Person/ Contacts | | .0049 | Per-act probability of acquiring HIV from an infected source,^5^ calibrated as: Mean of insertive/receptive anal intercourse (74.5/10K), insertive/receptive penile/vaginal intercourse (6/10K), and transmission risk through shared injection equipment (63/10K) = 47.83/10K; increased slightly because of higher incidence among MSM in the region. This number is being used in both high and low prevalence areas in the model. |
| Total UNDIAGNOSED and DIAGNOSED PLWH | auxiliary | People | | UNDIAGNOSED HIV Positive Persons + NEWLY DIAGNOSED Late HIV Testers Not in HIV Care + NEWLY DIAGNOSED Early HIV Testers Not in HIV Care + PLWH ENGAGED in HIV MEDICAL CARE + PLWH LOST TO HIV MEDICAL CARE | Total number of PLWH, including the estimated number of undiagnosed PLWH and PLWH in all stages of the HIV care continuum over time. |
| Proportion of HIV negative population on PrEP | auxiliary | dmnl | | HIV TESTING AND PREVENTION SERVICES.On PrEP / (Persons Living in High Prevalence Communities - Total UNDIAGNOSED and DIAGNOSED PLWH) | Proportion of HIV-negative people living in high prevalence communities in the catchment area who are on pre-exposure prophylaxis (PrEP). (The On PrEP variable is imported from the “HIV Testing & Prevention” module). |
| HIV infection rate in high prevalence communities | flow | Person/ Month | | Persons Living in High Prevalence Communities * (1 - Proportion of PLWH who are VS among all PLWH) * (1 - Proportion of HIV negative population on PrEP) * (Risky contacts per month in high prevalence areas) * (Per contact risk of HIV infection) {UNIFLOW} | Monthly rate of new HIV infections in high HIV prevalence communities over time. This rate is reduced by the proportion of HIV-negative people on PrEP and the proportion of PLWH who are virally suppressed (VS) in the high prevalence areas. |
| Prop of total population living in low prevalence communities | auxiliary | dmnl | | 1 - Prop of total population living in high prevalence communities | Estimated proportion of people living all communities in the catchment area (Hartford TGA) not designated as highest prevalence, that is, in cities and towns with <500/10K population who have HIV. |
| In migration to low prev communities | flow | Person/ Month | | Prop of total population living in low prevalence communities * In Migration {UNIFLOW} | Monthly rate of migration into the low HIV prevalence communities, applying the same in-migration rate as to the whole catchment area. |
| Persons Living in Low Prevalence Communities | stock | Persons | | Persons Living in Low Prevalence Communities(t - dt) + (In migration to low prev communities - Out migration from low prevalence communities - HIV infection rate in low prevalence communities) * dt {NON-NEGATIVE} Initial value = Initial total population in catchment area * Prop of total population living in low prevalence communities | Number of people in the Hartford TGA living in communities outside the highest prevalence cities in 2017 (<500/10K HIV-positive residents) over time. |
| **Variable Name** | **Variable Type** | **Unit** | **Initial Parameter Values and Formulas** | | **Variable Definition/Specification and**  **Sources of Initial Parameters and Stock Values** |
| Prop of total population experiencing low prevalence risk | auxiliary | dmnl | | Persons Living in Low Prevalence Communities / Total Population in Catchment Area | Proportion of residents living in low HIV prevalence communities over the total population of the catchment area over time. |
| Out migration from low prevalence communities | flow | Person/ Month | | Prop of total population experiencing low prevalence risk * Out Migration {UNIFLOW} | Monthly rate of migration out of the low HIV prevalence communities, applying the same out-migration rate as to the whole catchment area. |
| Risky contacts per month in low prevalence areas | auxiliary | Contacts/Person/ Month | | .003 | Estimated rate of HIV exposure per month of residents living in a low HIV prevalence community within the targeted catchment area. Determined via model calibration to reflect known historical HIV diagnosis rates for the total catchment area (8.25 persons/ month) and known difference in diagnosed cases of HIV in high to low prevalence communities (3:1 ratio). |
| HIV infection rate in low prevalence communities | flow | Person/ Month | | Persons Living in Low Prevalence Communities * (1 - Proportion of PLWH who are VS among all PLWH) * (1 - Proportion of HIV negative population on PrEP) * Risky contacts per month in low prevalence areas {UNIFLOW} | Monthly rate of new HIV infections in low HIV prevalence communities over time. This rate is reduced by the proportion of HIV-negative people on PrEP and the proportion of PLWH who are virally suppressed (VS) in the low prevalence areas. |
| **HIV Treatment as Prevention and Treatment Cascade** | | | | | |
| Cumulative HIV incidence | stock | stock | | Cumulative HIV incidence(t - dt) + (HIV inc) * dt {NON-NEGATIVE} Initial value = 0 | Total number of new HIV infections in the catchment area over time. |
| HIV inc | flow | Persons/ month | | HIV incidence rate {UNIFLOW} | Monthly rate of people in the catchment area (Hartford TGA) becoming HIV infected per month, equal to the HIV incidence rate (defined above). |
| Prop deaths of PLWH per month | auxiliary | Persons/Persons/Month | | .002 | Monthly proportion of deaths of PLWH (out of all diagnosed PLWH living in CT) from any cause. Based on CT DPH HIV Surveillance Division, Annual Mortality Rate of diagnosed PLWH.^4^ |
| HIV incidence rate | flow | Persons/ month | | HIV infection rate in high prevalence communities + HIV infection rate in low prevalence communities {UNIFLOW} | Monthly rate of people in the total catchment area becoming newly HIV infected over time (sum of high prevalence and low prevalence monthly incidence rates). |
| UNDIAGNOSED HIV Positive Persons | stock | Persons | | UNDIAGNOSED HIV Positive Persons(t - dt) + (HIV incidence rate - HIV early test pos rate - HIV late test pos rate - "Undiagnosed HIV+ mortality rate") * dt {NON-NEGATIVE} Initial value = Init UNDIAGNOSED | Number of undiagnosed PLWH over time, including all newly infected people prior to HIV diagnosis. |
| Undiagnosed HIV pos mortality rate | flow | Persons/ month | | UNDIAGNOSED HIV Positive Persons * Prop deaths of PLWH per month {UNIFLOW} | Monthly rate of undiagnosed PLWH who die of any cause before being diagnosed with HIV, using the CT DPH HIV Surveillance rate of HIV deaths per month. |
| Prop of late testers | auxiliary | dmnl | | 0.27 | Proportion of people infected with HIV in the catchment area (Hartford TGA) who tested late in the reporting period 2016 (ending 12/31/2016). Late testers are defined as being diagnosed with AIDS within 12 months of testing positive for HIV. CT DPH HIV Surveillance.^4^ |
| **Variable Name** | **Variable Type** | **Unit** | **Initial Parameter Values and Formulas** | | **Variable Definition/Specification and**  **Sources of Initial Parameters and Stock Values** |
| Est time to late test | auxiliary | Months | | UNDIAGNOSED HIV Positive Persons / HIV late test pos rate | This is a model-generated estimate of the time from infection to diagnosis for persons who are late testers (defined as diagnosed with AIDS concurrent with their HIV diagnosis or within 12 months of being diagnosed with HIV). |
| HIV late test pos rate | flow | Persons/ month | | HIV TESTING AND PREVENTION SERVICES.Total HIV positive tests per month * Prop of late testers {UNIFLOW} | Monthly rate of undiagnosed HIV-positive cases getting tested and diagnosed with HIV who are late testers. (Late testers are defined as being diagnosed with AIDS within 12 months of testing positive for HIV.) The HIV-positive tests per month variable is imported from the “HIV Testing & Prevention” module. |
| Est time to early test | auxiliary | Months | | UNDIAGNOSED HIV Positive Persons / HIV early test pos rate | This is a model-generated estimate of the time from infection to diagnosis for persons who are NOT late testers because they did not have an AIDS diagnosis concurrent with or within 12 months of their HIV diagnosis. |
| HIV early test pos rate | flow | Persons/ month | | HIV TESTING AND PREVENTION SERVICES.Total HIV positive tests per month * (1 - Prop of late testers) {UNIFLOW} | Monthly rate of undiagnosed HIV-positive cases getting tested and diagnosed with HIV who are NOT late testers, that is, they did not have an AIDS diagnosis within 12 months of their HIV diagnosis. The total number of HIV-positive tests per month is imported from the “HIV Testing & Prevention” module. |
| NEWLY DIAGNOSED Late HIV Testers Not In HIV Care | stock | Persons | | NEWLY DIAGNOSED Late HIV Testers Not in HIV Care(t - dt) + (HIV late test pos rate - Late Tester Linked to Care rate - Mortality rate Late HIV Testers) * dt {NON-NEGATIVE} Initial value = 0 | Number of newly diagnosed PLWH over time who are late testers (AIDS diagnosis within 12 months of HIV diagnosis) who are not yet linked to medical care. |
| Mortality rate Late HIV Testers | flow | Persons/ month | | NEWLY DIAGNOSED Late HIV Testers Not in HIV Care * Prop deaths of PLWH per month {UNIFLOW} | Monthly rate of newly diagnosed late HIV testing PLWH who die of any cause before being linked to medical care, using the CT DPH HIV Surveillance rate of HIV deaths per month.^4^ |
| Late Tester Link to Care rate | flow | Persons/ month | | MAX(("MEDICAL CARE SERVICES FOR PEOPLE LIVING WITH HIV (PLWH)".Linked to care rate * Prop of late testers), 0) {UNIFLOW} | Monthly rate of newly diagnosed late testing PLWH being linked to medical care for the first time. The Linked to care rate is being imported from the “Medical Care Services for PLWH” module. |
| NEWLY DIAGNOSED Early HIV Testers Not In HIV Care | stock | Persons | | NEWLY DIAGNOSED Early HIV Testers Not in HIV Care(t - dt) + (HIV early test pos rate - Early Tester Link to Care rate - Mortality rate Early HIV testers) * dt {NON-NEGATIVE} Initial value = 0 | Number of newly diagnosed PLWH over time who are NOT late testers (no AIDS diagnosis within 12 months of HIV diagnosis) who are not yet linked to medical care. |
| Mortality rate Early HIV testers | flow | Persons/ month | | NEWLY DIAGNOSED Early HIV Testers Not in HIV Care * Prop deaths of PLWH per month {UNIFLOW} | Monthly rate of newly diagnosed non-late HIV testing PLWH who die of any cause before being linked to medical care, using the CT DPH HIV Surveillance rate of HIV deaths per month. |
| Early Tester Link to Care rate | flow | Persons/ month | | MAX(("MEDICAL CARE SERVICES FOR PEOPLE LIVING WITH HIV (PLWH)".Linked to care rate * (1 - Prop of late testers)), 0) {UNIFLOW} | Monthly rate of newly diagnosed non-late testing PLWH being linked to medical care for the first time. The Linked to care rate is being imported from the “Medical Care Services for PLWH” module. |
| **Variable Name** | **Variable Type** | **Unit** | **Initial Parameter Values and Formulas** | | **Variable Definition/Specification and**  **Sources of Initial Parameters and Stock Values** |
| PLWH ENGAGED in HIV MEDICAL CARE | stock | Persons | | PLWH ENGAGED in HIV MEDICAL CARE(t - dt) + (Early Tester Link to Care rate + Return to care rate + Late Tester Link to Care rate - Lost to care rate - In care mort rate) * dt {NON-NEGATIVE}  Initial value = Init DIAGNOSED PLWH * Init prop ENGAGED IN CARE | Number of PLWH who are engaged in medical care over time. The initial value is defined as having reported CD4/VL counts to CT DPH within the 12 month reporting period in 2017 in the Hartford TGA.^4^ |
| In care mort rate | flow | Persons/ month | | PLWH ENGAGED in HIV MEDICAL CARE * Prop deaths of PLWH per month {UNIFLOW} | Monthly rate of PLWH engaged in medical care who die of any cause, using the CT DPH HIV Surveillance rate of HIV deaths per month. |
| Lost to care rate | flow | Persons/ month | | "MEDICAL CARE SERVICES FOR PEOPLE LIVING WITH HIV (PLWH)".Lost to care rate {UNIFLOW} | Monthly rate of PLWH becoming lost to care after having been linked to care for the first time. The Lost to care rate is imported from the “Medical Care Services for PLWH” module. |
| PLWH LOST TO HIV MEDICAL CARE | stock | Persons | | PLWH LOST TO HIV MEDICAL CARE(t - dt) + (Lost to care rate - Return to care rate - PLWH Lost to Care mortality rate) * dt {NON-NEGATIVE}  Initial value = Init DIAGNOSED PLWH * Init prop of DIAGNOSED PLWH LOST TO CARE | Number of PLWH who are lost to medical care over time. The initial value is defined as not having seen a medical provider with prescribing privileges in the 12-month reporting period. |
| Return to care rate | flow | Persons/ month | | MEDICAL CARE SERVICES FOR PEOPLE LIVING WITH HIV (PLWH)".Lost to care returning rate {UNIFLOW} | Monthly rate of PLWH who have been lost to care returning to the medical care of a prescribing doctor and updating VL/CD4 counts. The Return to care rate is imported from the “Medical Care Services for PLWH” module. |
| PLWH Lost to Care mortality rate | flow | Persons/ month | | PLWH LOST TO HIV MEDICAL CARE * Prop deaths of PLWH per month {UNIFLOW} | Monthly rate of PLWH who are lost to care who die of any cause, using the CT DPH HIV Surveillance rate of HIV deaths per month. |
| GOAL proportion on ART | auxiliary | dmnl | | 1.0 | Goal set by the community for the desired proportion of PLWH to be on antiretroviral treatment (ART), set at 100%, consistent with CT plan for "Getting to Zero" and treatment as prevention. |
| Expected number of PLWH USING ART | auxiliary | persons | | GOAL proportion on ART * PLWH ENGAGED in HIV MEDICAL CARE | Maximum number of PLWH who are expected to be on antiretroviral treatment given the community’s goal for the proportion on ART. |
| Time to start ART | auxiliary | Months | | 0.25 | Estimated time it takes for a newly diagnosed PLWH who has been linked to medical care to be prescribed and begin to take ART. CDC recommends initiating ART with all newly diagnosed PLWH immediately upon confirmed diagnosis with HIV. |
| Change in PLWH using ART | flow | Persons/ month | | (Expected number of PLWH USING ART - PLWH Engaged in Care using ART) / Time to start ART | Monthly rate at which PLWH who are engaged in HIV medical care initiate treatment and become patients on ART. This is affected by the estimated time it takes to begin ART after diagnosis with HIV. |
| PLWH Engaged in Care using ART | stock | persons | | PLWH Engaged in Care using ART(t - dt) + (Change in PLWH using ART) * dt {NON-NEGATIVE}  Initial value = Init DIAGNOSED PLWH * Init prop ENGAGED IN CARE * Init est prop of Diagnosed on ART | Number of PLWH who are on ART in the catchment area over time. |
| **Variable Name** | **Variable Type** | **Unit** | **Initial Parameter Values and Formulas** | | **Variable Definition/Specification and**  **Sources of Initial Parameters and Stock Values** |
| Prop of PLWH Engaged in Care who are ADHERENT to ART | auxiliary | dmnl | | 0.95 | Derived from the proportional difference between those reported to be engaged in care and those who were virally suppressed in the catchment area (Hartford TGA) by 12/31/2017. CT DPH, HIV Continuum of Care.^4^ Note: Affirming uncertainty in the true value of this parameter, sensitivity to changes in “prop of virally suppressed of diagnosed PLWH” relative to this proportion adherent to ART parameter was assessed (Range: .85 – 1.0). |
| Expected number of PLWH ACHIEVING SUPPRESSION | auxiliary | persons | | PLWH Engaged in Care using ART * Prop of PLWH Engaged in Care who are ADHERENT to ART | Calibration of how many PLWH on ART will achieve viral suppression based on the proportion who are adherent to their medication. |
| Time to achieve Viral Suppression | auxiliary | month | | 6 | Estimated time needed to achieve viral suppression after initiating ART medication.^6,7^ |
| Change in PLWH who are VS | flow | Persons/ month | | (Expected number of PLWH ACHIEVING SUPPRESSION - PLWH Engaged in Care who are VIRALLY SUPPRESSED) / Time to achieve Viral Suppression | Monthly rate of PLWH who are on ART and adherent to ART becoming virally suppressed. |
| PLWH Engaged in Care who are VIRALLY SUPPRESSED | stock | persons | | PLWH Engaged in Care who are VIRALLY SUPPRESSED(t - dt) + (Change in PLWH who are VS) * dt {NON-NEGATIVE}  Initial value = Init DIAGNOSED PLWH * Init prop VIRALLY SUPPRESSED | Number of PLWH who are virally suppressed in the catchment area over time. |
| **HIV Treatment as Prevention Outcomes** | | | | | |
| Proportion of PLWH who are VS among DIAGNOSED PLWH | auxiliary | dmnl | | PLWH Engaged in Care who are VIRALLY SUPPRESSED / (PLWH ENGAGED in HIV MEDICAL CARE + NEWLY DIAGNOSED Late HIV Testers Not in HIV Care + NEWLY DIAGNOSED Early HIV Testers Not in HIV Care + PLWH LOST TO HIV MEDICAL CARE) | Proportion of PLWH who are virally suppressed out of all diagnosed PLWH. (This dynamic proportion is comparable to CT DPH reported proportion of VS PLWH, CT DPH CT DPH, HIV Continuum of Care.)^4^ |
| Proportion of PLWH who are VS among all PLWH | auxiliary | dmnl | | PLWH Engaged in Care who are VIRALLY SUPPRESSED / (UNDIAGNOSED HIV Positive Persons + NEWLY DIAGNOSED Early HIV Testers Not in HIV Care + NEWLY DIAGNOSED Late HIV Testers Not in HIV Care + PLWH ENGAGED in HIV MEDICAL CARE + PLWH LOST TO HIV MEDICAL CARE) | Proportion of PLWH who are virally suppressed out of all PLWH, including diagnosed and undiagnosed. |

1. Song R, Hall HI, Green TA, Szwarcwald CL, Pantazis N. Using CD4 data to estimate HIV incidence, prevalence, and percent of undiagnosed infections in the United States. JAIDS Journal of Acquired Immune Deficiency Syndromes 2017;74:3-9.

2. CT Department of Public Health. Estimated HIV prevalence among persons aged >=13 years, by selected characteristics, Connecticut, 2016. In: Surveillance HA, ed. Hartford, CT 2018.

3. Connecticut HIV Statistics Through 2014. 2015. (Accessed April 15, 2016, at <http://www.ct.gov/dph/cwp/view.asp?a=3135&q=393048>.)

4. CT Department of Public Health. Epidemiological Profile of HIV in Connecticut. 2018.

5. Centers for Disease Control & Prevention. HIV Risk Behaviors: Estimated Per-Act Probability of Acquiring HIV from an Infected Source, by Exposure Act. December 2015.

6. Centers for Disease Control & Prevention. Evidence of HIV Treatment and Viral Suppression in Preventing the Sexual Transmission of HIV. In: National Center for HIV/AIDS VH, STD, and TB Prevention, Division of HIV/AIDS Prevention, ed.2018:1-5.

7. Cohen MS, Chen YQ, McCauley M, et al. Prevention of HIV-1 infection with early antiretroviral therapy. The New England journal of medicine 2011;365:493-505.
